# Safety and Efficacy of Transaortic Transcatheter Aortic Valve Implantation Via Right Mini-Thoracotomy

**DOI:** 10.1093/icvts/ivag226

**Published:** 2026-08-14

**Authors:** Naonori Kawamoto, Kizuku Yamashita, Kota Suzuki, Takashi Kakuta, Ayumi Ikuta, Rieko Kutuzawa, Satsuki Fukushima

**Affiliations:** Department of Cardiac Surgery, National Cerebral and Cardiovascular Center, Suita, Osaka 564-8565, Japan; Department of Cardiac Surgery, National Cerebral and Cardiovascular Center, Suita, Osaka 564-8565, Japan; Department of Cardiac Surgery, National Cerebral and Cardiovascular Center, Suita, Osaka 564-8565, Japan; Department of Cardiac Surgery, National Cerebral and Cardiovascular Center, Suita, Osaka 564-8565, Japan; Department of Cardiac Surgery, National Cerebral and Cardiovascular Center, Suita, Osaka 564-8565, Japan; Department of Cardiac Surgery, National Cerebral and Cardiovascular Center, Suita, Osaka 564-8565, Japan; Department of Cardiac Surgery, National Cerebral and Cardiovascular Center, Suita, Osaka 564-8565, Japan

**Keywords:** right mini-thoracotomy, transaortic transcatheter aortic valve implantation, transfemoral transcatheter aortic valve replacement, propensity score-matched analysis

## Abstract

**Objectives:**

Transcatheter aortic valve implantation (TAVI) via the transfemoral (TF) approach is widely used. However, patients with unsuitable peripheral arterial anatomy require alternative transthoracic access. In such patients, the transaortic TAVR via right mini-thoracotomy (Rt-TAo) approach offers a less invasive option than conventional sternotomy. Therefore, this study aimed to evaluate the procedural and 2-year clinical outcomes of TF TAVI versus Rt-TAo TAVI.

**Methods:**

Patients who underwent TAVI via the TF approach or the Rt-TAo approach between January 2012 and November 2023 were retrospectively reviewed. Baseline characteristics and clinical outcomes were compared, and propensity score matching was performed to adjust for baseline differences. The primary end-points were in-hospital mortality, stroke, other periprocedural morbidities, and postprocedural length of hospitalization. Secondary end-points included overall survival, freedom from cardiac-related death, and major adverse cardiac and cerebrovascular events (MACCE) at 2 years.

**Results:**

A total of 767 consecutive patients were included in the analysis, of whom 694 underwent TF TAVI and 73 underwent Rt-TAo TAVI. Before matching, the Rt-TAo group had higher baseline prevalences of comorbidities and Society of Thoracic Surgeon risk scores. After matching, the Rt-TAo group had a longer procedural time, higher transfusion requirements, and longer lengths of intensive care unit and total hospital stays. However, the 2 groups had similar rates of periprocedural stroke, coronary obstruction, annular rupture, access-related complications, and in-hospital mortality. The 2 groups had similar 2-year rates of survival and freedom from MACCE.

**Conclusions:**

The Rt-TAo TAVI is a safe and feasible alternative for patients with unsuitable peripheral arterial access, demonstrating comparable 2-year outcomes to TF TAVI.

## Graphical abstract

**Figure:**
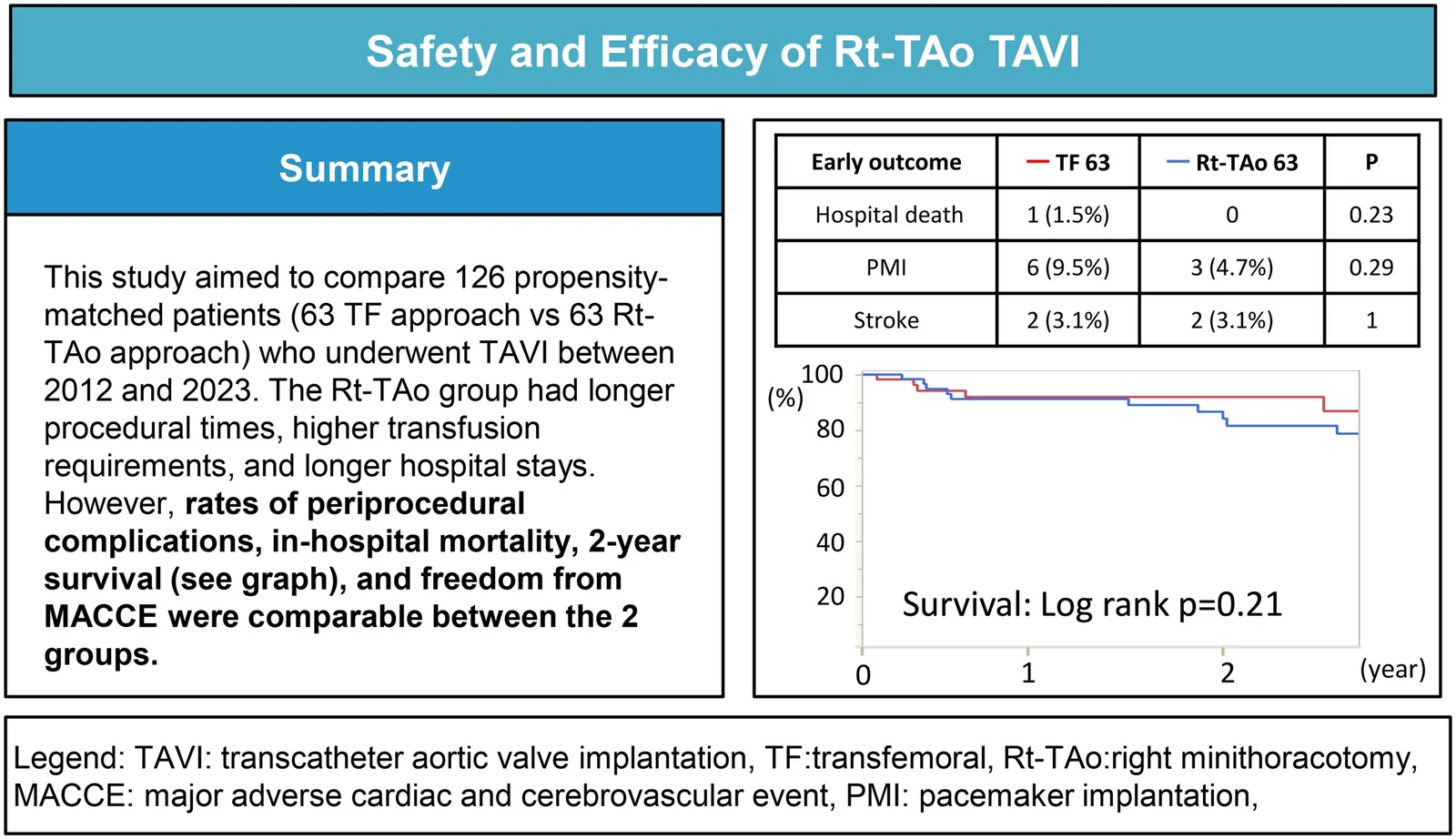

## INTRODUCTION

Transcatheter aortic valve implantation (TAVI) is a less invasive treatment for severe aortic stenosis, and outcomes are influenced by the access route. The transfemoral (TF) approach is the standard route because of its simplicity and safety profile. However, in patients with unsuitable peripheral arterial access (iliofemoral artery, subclavian artery, and carotid artery access), alternative transthoracic routes such as the transaortic (TAo) or transapical approach may be used.^1^^,^^2^

Previous studies indicate that the transapical approach has worse outcomes than the TF approach.^3–5^ Furthermore, the safety and feasibility of the TAo approach as an alternative route with good short-term outcomes have been demonstrated in several retrospective studies and a multicentre prospective study.^4^^,^^6–11^ However, the clinical outcomes of TAVI via the TF and transaortic via right mini-thoracotomy (Rt-TAo) approaches, which are less invasive alternatives to partial sternotomy,^12^ remain inadequately characterized. Therefore, this study aimed to compare the clinical outcomes of TAVI performed via the TF and Rt-TAo approaches, focusing on procedural safety, efficacy, and complication profiles.

## METHODS

### Study design and patient population

This was a single-centre retrospective observational study. We retrospectively reviewed the surgical database of the National Cerebral and Cardiovascular Center and identified patients who underwent TAVI between January 2012 and November 2023. Baseline demographic characteristics, comorbidities, preoperative echocardiographic parameters, procedural characteristics, and follow-up clinical outcomes were obtained from the institutional database and verified through electronic medical records. Follow-up information was supplemented by telephone interviews with patients or referring physicians when follow-up was performed at outside institutions. Patients who could not be contacted after their last documented follow-up were considered lost to follow-up and were censored at the date of last contact.

The TF approach was the primary access route, selected according to iliofemoral vessel size, calcification, and tortuosity. The Rt-TAo approach was considered in patients with iliofemoral artery diameter < 5 mm, severe iliofemoral artery stenosis or occlusion, a shaggy aorta, severe vascular calcification, or marked arterial tortuosity or kinking.

All patients were discussed by a multidisciplinary Heart Team consisting of interventional cardiologists, cardiovascular surgeons, cardiac anaesthesiologists, and imaging specialists. The Heart Team determined patient eligibility, prosthesis selection, and the most appropriate access route based on individual clinical and anatomical characteristics. Transcatheter aortic valve implantation procedures were subsequently performed collaboratively by interventional cardiologists and cardiovascular surgeons.

### Standardized Rt-TAo TAVI protocol

**Figure 1.**
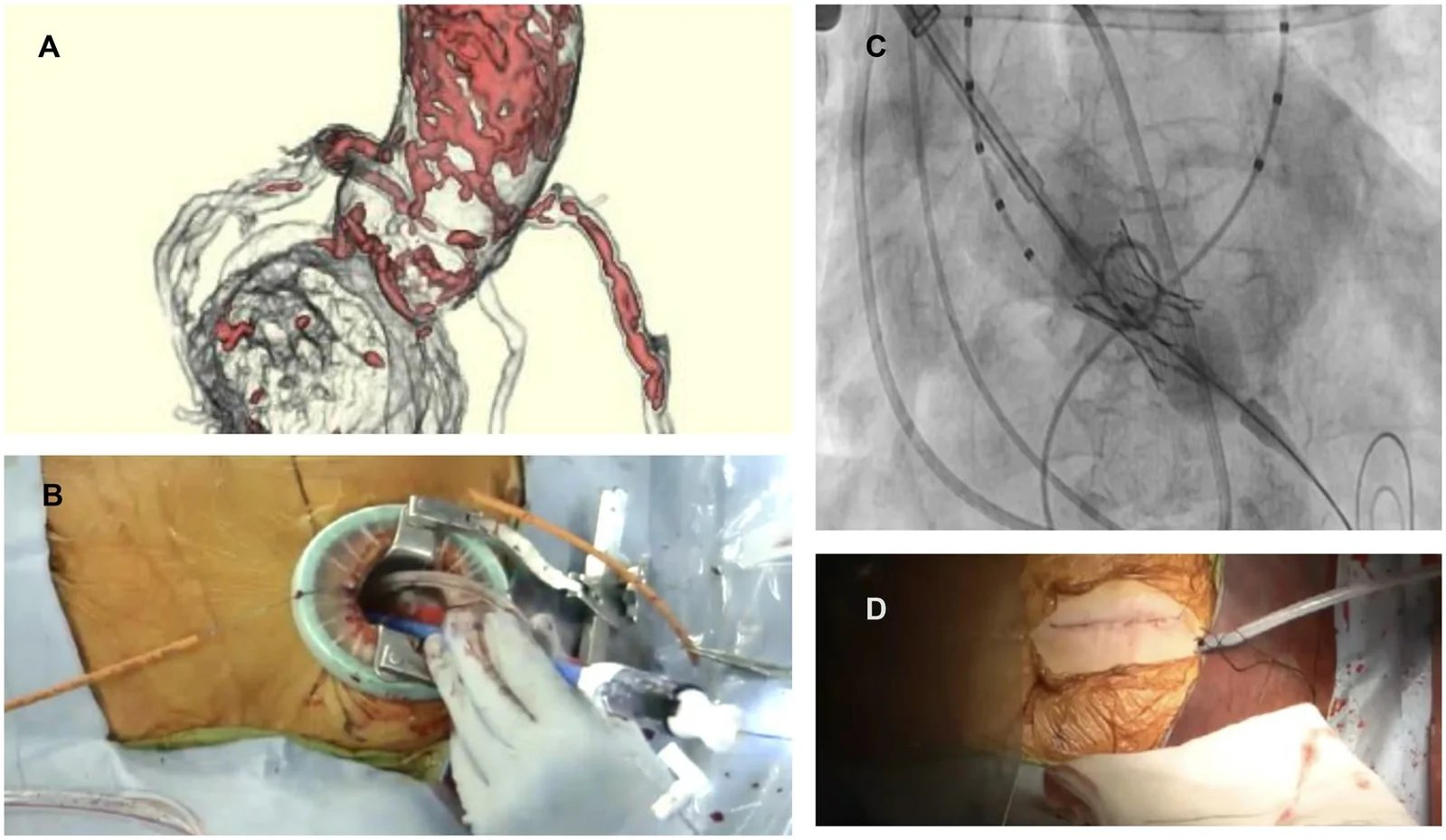
Main Steps of Rt-TAo TAVI. (A) Preprocedural assessment. Circumferential calcification at the intended ascending aortic puncture site was considered a contraindication to the Rt-TAo approach. (B) Manipulation of the ascending aorta. The ascending aorta was exposed through the second or third intercostal space. Delivery sheath was inserted into the ascending aorta. (C) Valve delivery and deployment. The transcatheter heart valve was advanced across the native aortic valve and deployed under fluoroscopic and transoesophageal echocardiographic guidance. (D) Haemostasis and closure. A single pleural chest tube was inserted before wound closure

Enhanced CT was used to perform preprocedural assessments of the calcification in the ascending aorta and the access-site anatomy. Circumferential or bulky calcification at the intended ascending aortic puncture site was considered a contraindication to the Rt-TAo approach (**Figure 1A**). Ascending aortic enlargement (diameter >40 mm) was regarded as a relative contraindication because it may compromise procedural safety and sheath positioning. However, the final decision regarding suitability for Rt-TAo access was made by the operating surgeon after consideration of the patient’s overall clinical and anatomical characteristics and the balance between procedural risks and benefits. For procedural safety, the ascending aorta was preferably located to the right of the sternal border at the second or third intercostal space. Details of the Rt-TAo TAVI procedure are described below.

Right mini-thoracotomy was performed through the second or third intercostal space (**Figure 1B**). A 5-cm skin incision was created from the right sternal border. The right internal mammary vessels were either retracted or ligated. The pericardium was incised and retracted with stay sutures to expose the ascending aorta. A soft-tissue retractor or small rib spreader was used to support the exposure of the ascending aorta. Heparin was administered to achieve an activated clotting time of 300 seconds. After insertion of a scaled pigtail catheter through the femoral artery or upper-extremity artery used for angiographic guidance, the site for the purse-string sutures on the ascending aorta was identified, and 2 circular purse strings (4-0 polypropylene) were placed at the selected spot. The sutures were secured with snares.

During aortic manipulation from the needle puncture to the removal of the delivery sheath, systolic blood pressure was continuously maintained below 100 mmHg. The aortic valve was crossed using a standard catheter, which was subsequently exchanged for an extra-stiff guidewire positioned in the left ventricle. An appropriately sized delivery sheath was then advanced over the guidewire into the ascending aorta. Balloon aortic valvuloplasty was performed at the operator’s discretion under rapid ventricular pacing. The transcatheter heart valve (THV) was advanced across the native aortic valve and positioned under fluoroscopic and transoesophageal echocardiographic guidance to achieve optimal coaxial alignment with the aortic annulus. After confirmation of valve position, the prosthesis was deployed according to the manufacturer’s instructions (**Figure 1C**). The valve function, position, paravalvular regurgitation, and coronary flow were assessed by transoesophageal echocardiography.

Under rapid pacing with the systolic blood pressure lowered to less than 30-40 mmHg, the delivery sheath was removed, and the purse strings were tied. Protamine was administered to reverse the heparin. One pleural chest tube was inserted, and the incision was closed after haemostasis (**Figure 1D**). All aortic procedures were performed by board-certified cardiovascular surgeons.

No cerebral embolic protection devices were used during the study period because these devices were not approved for reimbursement in Japan.

### Endpoints

The primary end-points were the rates of in-hospital mortality, stroke, and other periprocedural morbidities, and the postprocedural length of hospital stay. The secondary end-points were 2-year survival, freedom from cardiac death, and freedom from major adverse cardiac and cerebrovascular events (MACCE) were defined as a composite end-point comprising any of the following adverse events: death from any cause, stroke, myocardial infarction, and coronary intervention.

### Ethics statement

This study was conducted in accordance with the ethical principles of the Declaration of Helsinki. The Institutional Review Board of the National Cerebral and Cardiovascular Center approved the study protocol (approval number: M30-026-11; approval date: March 25, 2022). Written informed consent was obtained from all patients prior to study participation. No biological specimens were collected or stored for future research purposes.

### Propensity score-matched analysis

Because the baseline characteristics differed between the TF TAVI and Rt-TAo TAVI groups, a propensity score-matched analysis was performed to compare the early and 2-year outcomes between the 2 groups. Propensity scores were calculated through multiple logistic regression using the following 20 variables: sex, age, body surface area, New York Heart Association class, hypertension, hyperlipidaemia, cerebrovascular event, atrial fibrillation, arteriosclerosis obliterans, coronary artery disease, chronic kidney disease requiring haemodialysis, elective surgery, diabetes mellitus, chronic lung disease, bicuspid aortic valve, history of percutaneous coronary intervention, preoperative ejection fraction, peak aortic valve velocity, Society of Thoracic Surgeons risk score, and frailty scale. One-to-one nearest-neighbour matching without replacement was performed using a caliper width of 0.2 times the standard deviation of the logit of the propensity score. The baseline characteristics of the total and matched cohorts are listed in **Table 1**. Standardized differences were used to evaluate the baseline variables. C statistics were calculated to evaluate the goodness of fit of this model. Missing data accounted for less than 5% of all study variables. Patients with missing data for variables included in the propensity score model were excluded from the propensity score analysis. Given the low frequency of missing data, no imputation procedures were performed.

**Table 1.** Patient Characteristics in the TF and Rt-TAo TAVI

	Total cohort				Matched cohort			
	TF	Rt-TAo	SD	*P* value	TF	Rt-TAo	SD	*P* value
	*n* = 694	*n* = 73			*n* = 63	*n* = 63		
Age	83.8 ± 5.8	83.8 ± 6.7	0.008	.79	83.8 ± 5.3	84.1 ± 6.7	0.17	.54
Male sex	214 (30.8%)	28 (38.3%)	0.15	.19	32 (50.7%)	24 (38%)	0.13	.15
BSA	1.46 ± 0.17	1.45 ± 0.18	0.03	.66	1.49 ± 0.19	1.46 ± 0.18	0.018	.195
DM	136 (19.6%)	20 (27.4%)	0.18	.12	20 (31.7%)	16 (25.3%)	0	.42
ASO	18 (2.6%)	16 (21.9%)	0.61	<.0001	6 (10.5%)	6 (10.5%)	0	1.0
CAD	150 (21.6%)	33 (45.2%)	0.51	<.0001	26 (41.2%)	26 (41.2%)	0.06	1.0
STS risk score	6.4 ± 4.3	9.4 ± 6.3	0.53	<.0001	7.7 ± 5.1	8.5 ± 5.3	0.04	.28
Previous PCI	81 (11.6%)	16 (21.9%)	0.27	.019	9 (14.2%)	12 (19%)	0	.47
Haemodialysis	54 (7.7%)	12 (16.4%)	0.26	.02	10 (15.8%)	9 (14.2%)	0.11	.8
Pre-TAVR EF (%)	59 ± 10.7	54.2 ± 15.1	0.34	.02	57 ± 12.3	56 ± 13.2	0.089	.77
Pre-TAVR NYHA class	2.1 ± 0.5	2.4 ± 0.7	0.24	.06	2.3 ± 0.6	2.3 ± 0.7	0.16	.65
Peak AV velocity (m/s)	4.5 ± 0.7	4.3 ± 0.6	0.21	.07	4.3 ± 0.6	4.4 ± 0.7	0.03	.28
Urgent/emergency surgery	26 (3.75%)	6.8 (22%)	0.18	.1	4 (6.3%)	5 (7.9%)	0	.72
Lung disease	128 (18.4%)	9 (12.3%)	0.17	.17	6 (10.5%)	7 (11.1%)	0.11	.76
Afib/pAf	123 (17.7%)	17 (23.3%)	0.13	.25	12 (19%)	15 (23.8%)	0.07	.5
Frailty scale	3.8 ± 1.2	4.0 ± 1.3	0.12	.37	3.5 ± 0.9	4.1 ± 1.3	0.01	.09
Tricuspid valve	609 (94%)	65 (94%)	0%	.98	57 (98%)	55 (93%)	0.14	.17
History of CV event	110 (15.8%)	12 (16.4%)	0.015	.9	10 (15.8%)	11 (17.4%)	0.08	.8

Abbreviations: Afib, atrial fibrillation; ASO, arteriosclerosis obliterans; AV, aortic valve; BSA, body surface area; CAD, coronary artery disease; CV, cerebrovascular; DM, diabetes mellitus; EF, ejection fraction; NYHA, New York Heart Association; pAf, paroxysmal atrial fibrillation; PCI, percutaneous coronary intervention; Rt-TAo, transaortic approach through right mini-thoracotomy; SD, standardized difference; STS, Society of Thoracic Surgeons; TAVI, transcatheter aortic valve implantation; TF, transfemoral approach.

### Statistical analysis

Categorical data are presented as proportions, while continuous data are expressed as mean ± standard deviation. Differences between groups were assessed using the chi-squared test for categorical variables, the independent Student’s t-test for normally distributed continuous variables, and the Mann-Whitney *U*-test for non-normally distributed continuous variables. The rates of survival, freedom from reoperation, and freedom from MACCE were obtained using the Kaplan-Meier method and compared between groups using the log-rank test. *P* ≤ 0.05 was considered statistically significant. All statistical analyses were performed using JMP 18 (SAS Institute, Cary, NC, United States).

## RESULTS

### Baseline characteristics

The surgical database of the National Cerebral and Cardiovascular Center includes data on 844 consecutive patients who underwent TAVI between January 2012 and November 2023 at our centre. Patients who underwent transapical access (*n* = 58), subclavian access (*n* = 15), and transaortic access via sternotomy (*n* = 4) were excluded from the study cohort.

Consequently, a total of 767 consecutive patients were included in the analysis. The TF approach was performed in 694 patients (214 males [30.8%], mean age 83.8 years), and Rt-TAo TAVI was performed in 73 patients (28 males [38.3%], mean age 83.8 years). The mean follow-up period was 2.1 years in the TF TAVI group and 2.3 years in the Rt-TAo TAVI group. During the follow-up period, 39 patients (5%) were lost to follow-up. Follow-up completeness was 95%.

The baseline characteristics of the 2 groups in the total and matched cohorts are summarized in **Table 1**. In the total cohort, patients who underwent Rt-TAo TAVI were more likely to have had prior percutaneous coronary intervention (*P* = .019), arteriosclerosis obliterans (*P* < .001), haemodialysis (*P* = .02), and coronary artery disease (*P* < .001) compared with patients who underwent TF TAVI. Consequently, the Society of Thoracic Surgeons risk score was significantly higher in the Rt-TAo TAVI group than in the TF TAVI group in the total cohort (*P* < .001). Additionally, the preoperative ejection fraction was significantly lower in the Rt-TAo TAVI group than in the TF TAVI group in the total cohort (*P* = .02).

### Periprocedural and in-hospital outcomes

The periprocedural and in-hospital outcomes are summarized in **Tables 2**  **and**  **3**. The distribution of THV type and valve sizes was comparable between the TF and Rt-TAo groups. No significant differences were observed in the proportion of patients receiving prostheses ≤23 mm, either before or after propensity score matching. In the total cohort, the Rt-TAo TAVI group had a longer procedural time (*P* < .001) and a higher rate of balloon aortic valvuloplasty before valve deployment (*P* = .002) than the TF TAVI group. However, after matching, the 2 groups had a similar rate of balloon aortic valvuloplasty before valve deployment (*P* = .25), and the rate of post-TAVI balloon dilatation tended to be higher in the Rt-TAo TAVI group than in the TF TAVI group, without reaching statistical significance (*P* = .1). In the matched cohort, Rt-TAo TAVI was associated with a higher risk of transfusion than TF TAVI (*P* < .0001). The 2 matched groups had similar incidences of coronary occlusion, annulus rupture, left ventricular rupture, and access route injury. In the matched cohort, the TF approach resulted in a shorter intensive care unit stay (*P* = .003) and hospital length of stay (*P* = .0007) than the Rt-TAo approach. Furthermore, periprocedural stroke occurred in 3.1% of patients in both matched groups. In the unmatched cohort, 6 in-hospital deaths occurred in the TF TAVI group, whereas no in-hospital deaths occurred in the Rt-TAo TAVI group. The causes of death are summarized in **Table 3**. Two patients in the TF-TAVI group developed access-related aortic dissections, and both had undergone implantation of a self-expanding valve. One of these events occurred in the matched cohort and resulted in in-hospital mortality.

**Table 2.** Surgical Outcomes of TF and Rt-TAo TAVI

	Total cohort			Matched cohort		
	TF	Rt-TAo	*P* value	TF	Rt-TAo	*P* value
	*n* = 694	*n* = 73		*n* = 63	*n* = 63	
Transfusion	206 (29.6％)	57 (80.2%)	<.0001	25 (39％)	48 (78％)	<.0001
TAV in SAV	48 (6.9％)	4 (5.5％)	.63	5 (7.9％)	4 (6.4％)	.72
THV type						
BEV	427 (61.5%)	47 (64%)	.6	45 (71%)	41 (65%)	.56
SAPIEN 3 Ultra	58 (8%)	0		7 (11.1%)	0	
SAPIEN XT	70 (10%)	2 (2.7%)		3 (4.8%)	1 (1.6%)	
SAPIEN 3	299 (43.5%)	45 (61.6%)		35 (55.6%)	40 (63.5%)	
SEV	267 (38.5%)	26 (36%)	.6	18 (29%)	22 (35%)	.56
CoreValve	29 (4.2%)	6 (8.2%)		2 (3.2%)	3 (4.8%)	
Evolut R	86 (12.4%)	7 (9.6%)		4 (6.3%)	7 (11.1%)	
Evolut PRO	43 (6.2%)	4 (5.5%)		2 (3.2%)	4 (6.3%)	
Evolut PRO+	79 (11.4%)	6 (8.2%)		8 (12.7%)	6 (9.5%)	
Evolut FX	20 (2.9%)	1 (1.4%)		1 (1.6%)	1 (1.6%)	
Navitor	10 (1.4%)	2 (2.7%)		1 (1.6%)	1 (1.6%)	
THV size 23 mm or less	350 (50%)	34 (46%)	.53	29 (46%)	29 (46%)	1.0
Operation time (min)	92 ± 48	118 ± 41	<.0001	94 ± 54	118 ± 43	.0092
Coronary protection	11 (1.6%)	2 (2.8%)	.47	2 (3.2%)	2 (3.2%)	1.0
Pre-TAVI BAV	317 (45%)	20 (27%)	.002	23 (36%)	17 (27%)	.25
Post-TAVI BAV	106 (15%)	14 (19%)	.35	5 (7.9%)	11 (17.7%)	.1
Annulus rupture	1 (0.14%)	0	.65	0	0	1.0
Access route injury	20 (2.8%)	1 (1.3%)	.45	1 (1.5%)	1 (1.5%)	1.0
Coronary obstruction	7 (1.0%)	0	.23	1 (1.5%)	0	.23
Valve migration	5 (0.7%)	1 (1.3%)	.45	0	0	1.0

Abbreviations: BAV, balloon aortic valvuloplasty; BEV, balloon-expandable valve; Rt-TAo, transaortic approach through right mini-thoracotomy; SEV, self-expandable valve; TAVI, transcatheter aortic valve implantation; TAV in SAV, transcatheter aortic valve in surgical aortic valve replacement; TF, transfemoral approach; THV, transcatheter heart valve.

**Table 3.** In-Hospital Outcomes of TF and Rt-TAo TAVI

	Total cohort			Matched cohort		
	TF	Rt-TAo	*P* value	TF	Rt-TAo	*P* value
	*n* = 694	*n* = 73		*n* = 63	*n* = 63	
In-hospital death	6 (0.86%)	0	.27	1 (1.5%)	0	.23
Cause of in-hospital death						
Pneumonia	2 (0.43%)	0	.58	0	0	1.0
Access-related aortic dissection	2 (0.43%)	0	.58	1 (1.5%)	0	.23
Cerebrovascular event	2 (0.43%)	0	.58	0	0	1.0
PMI	53 (7.6%)	5 (6.8%)	.8	6 (9.5%)	3 (4.7%)	.29
Stroke	11 (1.59%)	2 (2.74%)	.5	2 (3.1%)	2 (3.1%)	1.0
Embolic event	10 (1.4%)	2 (2.7%)	.36	2 (3.1%)	2 (3.1%)	1.0
Respiratory failure	3 (0.4%)	3 (4.1%)	.001	1 (1.5%)	2 (3.1%)	.55
PVL ≥ moderate	15 (2.1%)	1 (1.3%)	.4	1 (1.5%)	1 (1.5%)	1.0
ICU length of stay (days)	2.7 ± 1.5	3.9 ± 2.9	<.0001	2.7 ± 0.2	3.8 ± 0.3	.003
Hospital length of stay (days)	10.8 ± 0.86	19.1 ± 3.1	<.0001	12.9 ± 2.1	15.9 ± 1.5	.0007

Abbreviations: AKI, acute kidney injury; ICU, intensive care unit; PMI, pacemaker implantation; PVL, perivalvular leakage; Rt-TAo, transaortic approach through right mini-thoracotomy; TAVI, transcatheter aortic valve implantation; TF, transfemoral approach.

### Survival, freedom from cardiac death, and freedom from MACCE at 2 years

**Figure 2.**
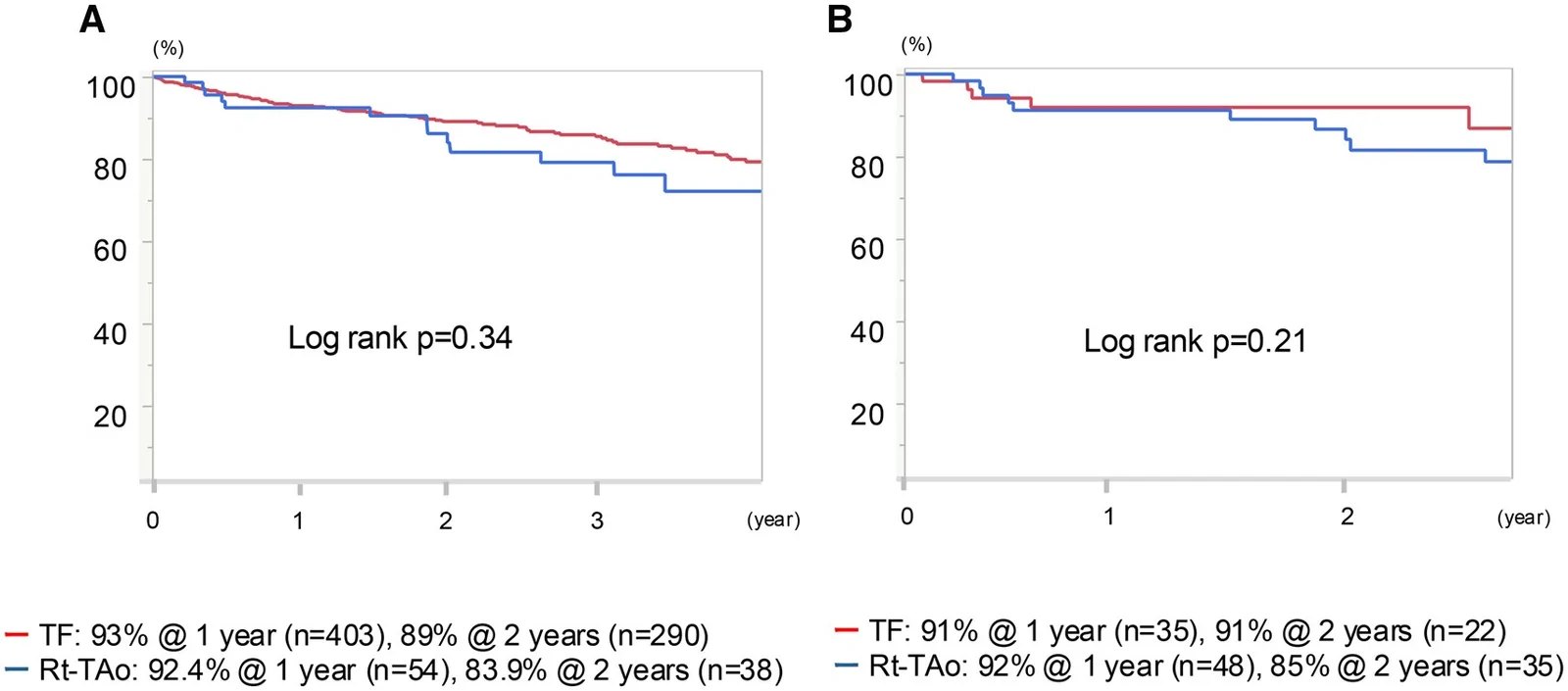
Survival Rates After Transcatheter Aortic Valve Replacement Via the Transfemoral Approach (TF TAVI) and the Transaortic via Right Mini-Thoracotomy Approach (Rt-TAo TAVI). The 2-year rates of survival after TF TAVI and Rt-TAo TAVI are (A) 89% and 84%, respectively, in the total cohort (log rank *P* = .34), and (B) 91% and 85%, respectively, in the matched cohort (log rank *P* = .21)

**Figure 3.**
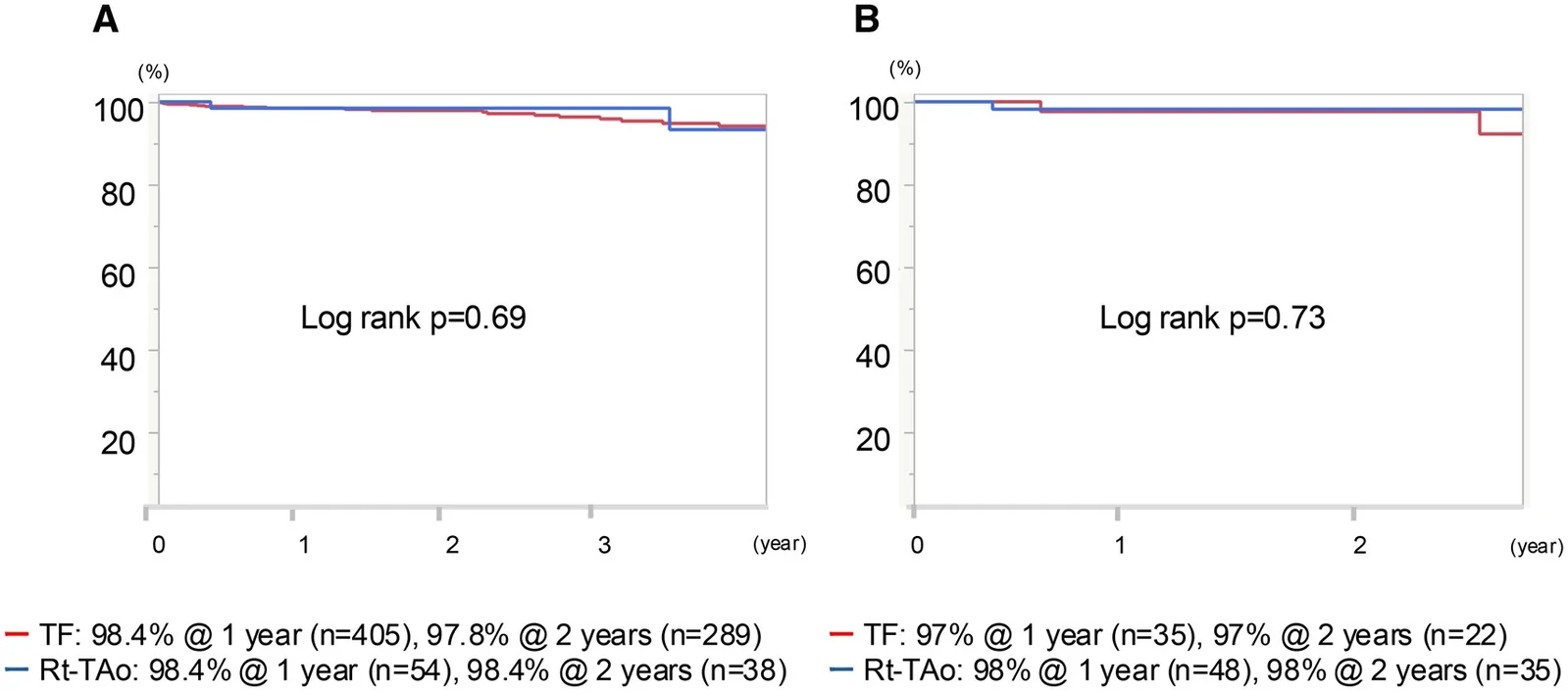
Rates of Freedom From Cardiac-Related Death After Transcatheter Aortic Valve Implantation Via the Transfemoral Approach (TF-TAVI) and the Transaortic via Right Mini-Thoracotomy Approach (Rt-TAo TAVI). The 2-year rates of freedom from cardiac-related death after TF TAVI and Rt-TAo TAVI are (A) 97.8% and 98.4%, respectively, in the total cohort (log rank *P* = .69), and (B) 97% and 98%, respectively, in the matched cohort (log rank *P* = .73)

**Figure 4.**
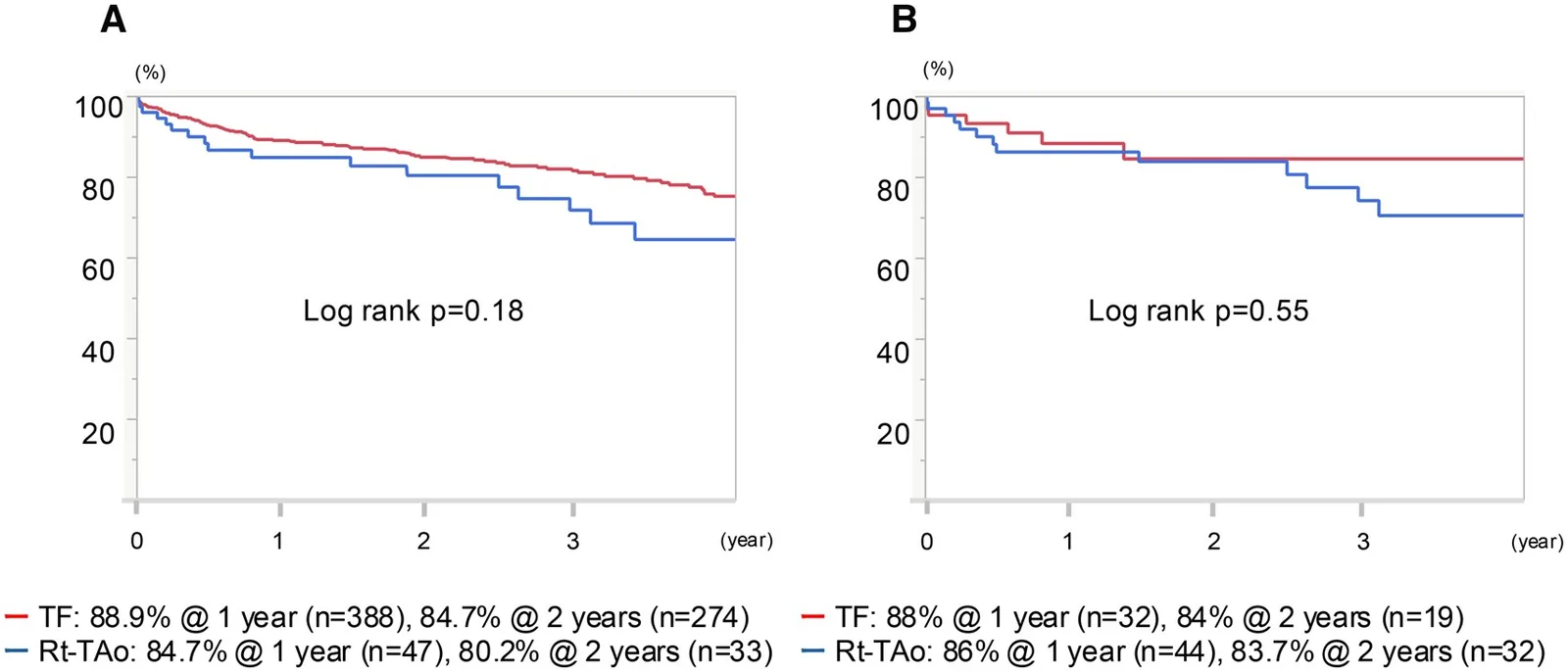
Rates of Freedom From Major Adverse Cardiac and Cerebrovascular Events (MACCE) After Transcatheter Aortic Valve Replacement Via the Transfemoral Approach (TF TAVI) and the Transaortic via Right Mini-Thoracotomy Approach (Rt-TAo TAVI). The 2-year rates of freedom from MACCE after TF TAVI and Rt-TAo TAVI are (A) 84.7% and 80.2%, respectively, in the total cohort (log rank *P* = .18), and (B) 84% and 83.7%, respectively, in the matched cohort (log rank *P* = .55)

In the total cohort, the 2-year survival rate was 89% after TF TAVI and 84% after Rt-TAo TAVI (log rank *P* = .34; **Figure 2A**). After matching, the 2-year survival rate was 91% after TF TAVI and 85% after Rt-TAo TAVI (log rank *P* = .21; **Figure 2B**). The causes of death during follow-up are summarized in **Table S1**. There were no significant differences between the 2 groups in the 2-year rates of freedom from cardiac death in either cohort (**Figure 3A and B**). In the total cohort, the 2-year rates of freedom from MACCE were 84.7% after TF TAVI and 80.2% after Rt-TAo TAVI (log rank *P* = .18; **Figure 4A**). After matching, the 2-year rates of freedom from MACCE were 84% after TF TAVI and 83% after Rt-TAo TAVI (*P* = .55; **Figure 4B**).

## DISCUSSION

Despite ongoing miniaturization of delivery systems, some Japanese patients remain unsuitable for peripheral-access TAVI because of small vessel diameters. Patients with a shaggy aorta or severe vascular calcification also require alternative access strategies.^1^^,^^2^

Several meta-analyses have reported higher early mortality with transapical than TF TAVI.^13–16^ In contrast, TAo and TF TAVI showed comparable early and follow-up survival.^10^ Therefore, the optimal non-peripheral alternative access route remains controversial.

Although significant differences in baseline characteristics, including the prevalence of peripheral arterial disease and haemodialysis, were present in the unmatched cohorts, these differences were well balanced after propensity score matching. After propensity score matching, there were no significant differences between the 2 groups in in-hospital mortality, periprocedural complications, 2-year survival, or 2-year freedom from MACCE.

The transaortic approach offers several advantages compared with other alternative access routes, including high procedural feasibility and direct control of valve positioning during deployment.^11^^,^^17^ In addition, TAo access allows the use of mechanical circulatory support devices—such as extracorporeal membrane oxygenation, intra-aortic balloon pump support, and the Impella system—via the peripheral arteries, even when those arteries are too small to accommodate TAVI delivery systems.

Upper partial sternotomy has traditionally been more commonly used than right anterior mini-thoracotomy for TAo TAVI.^10^^,^^11^^,^^18^ However, to date, no studies have directly compared partial sternotomy and anterior mini-thoracotomy specifically as access routes for TAo TAVI. Several studies comparing these approaches in the setting of minimally invasive surgical aortic valve replacement (MICS AVR) have suggested that anterior mini-thoracotomy may facilitate postoperative recovery by avoiding sternal division. In these studies, anterior mini-thoracotomy was associated with earlier postoperative mobilization and shorter hospital stay, while maintaining comparable early clinical outcomes, including 30-day mortality.^12^^,^^19^ Although these findings were derived from surgical AVR populations, they may be particularly relevant for elderly patients undergoing TAVI, in whom rapid recovery, early ambulation, and shorter hospitalization are important determinants of postoperative outcomes and quality of life.

From the perspective of non-femoral alternative access, transcarotid (TC) TAVI is currently the most frequently employed approach. However, TC TAVI requires temporary carotid artery clamping, raising concerns regarding cerebral hypoperfusion, especially in patients with limited collateral cerebral circulation. In contrast, the TAo approach avoids carotid artery manipulation and eliminates the need for carotid clamping, thereby reducing concerns related to cerebral ischaemia. Moreover, cerebral embolic protection devices cannot be used during TC TAVI, whereas their use is technically feasible during TAo TAVI. This distinction may be particularly important in patients with extensive aortic atherosclerosis, including those with a shaggy aorta, in whom the risk of cerebral embolization is high.

Furthermore, previous studies suggest that the Rt-TAo approach provides a more coaxial trajectory for valve deployment compared with partial sternotomy. Patients with a horizontal aorta may benefit from right anterior thoracotomy access for TAVI.^20^ In the present study, Rt-TAo TAVI achieved 2-year outcomes comparable to those of TF TAVI, supporting the safety and efficacy of the Rt-TAo approach as an alternative access route in patients with severe peripheral arterial disease or hostile aortic anatomy. Importantly, no deaths in the Rt-TAo TAVI group were directly related to the access route or implanted valve. These findings suggest that the Rt-TAo approach can be performed safely when a standardized procedural protocol is applied by experienced, board-certified cardiovascular surgeons and interventional cardiologists. In contrast, 2 access-related aortic dissections occurred in the TF TAVI group, one of which resulted in in-hospital mortality. Both dissections occurred in patients treated with self-expanding valve systems. In contrast to earlier reports suggesting higher stroke rates with alternative access routes,^20^^,^^21^ we observed similar incidences of stroke between TF TAVI and Rt-TAo TAVI. These findings suggest that alternative access strategies, including the Rt-TAo approach, are being appropriately selected for patients in whom TF access is not feasible.

### Limitations

First, this was a retrospective observational study conducted at a single centre and was limited by potential bias introduced by specific referral patterns at our institution. Furthermore, some variables, including patient selection criteria and institutional or Heart Team preference for Rt-TAo access, could not be fully accounted for and may have introduced residual confounding despite propensity score matching. Second, the follow-up period was short. Therefore, longer follow-up is needed to further evaluate the impact of the Rt-TAo approach on long-term outcomes. Third, causes of death were determined based on available clinical records, imaging findings, and information obtained from treating physicians. No postmortem examinations were performed, and therefore the precise causes of death could not be independently confirmed in all cases.

## CONCLUSIONS

Rt-TAo TAVI provides a viable alternative to TF TAVI in patients with unfavourable peripheral arterial anatomy. Although the Rt-TAo approach is associated with a longer procedure time, higher transfusion rates, and longer hospitalization, it provides comparable in-hospital and 2-year outcomes (including rates of mortality, stroke, and MACCE) to the TF approach after adjusting for baseline characteristics. These findings support the use of Rt-TAo TAVI as an effective, less invasive option when TF access is not feasible, emphasizing the importance of patient-specific access planning in TAVI.

## Supplementary Material

ivag226_Supplementary_Data

## Data Availability

The deidentified individual participant data underlying the findings of this study, including data dictionaries, are available from the corresponding author upon reasonable request.
