# Supplementary material for: Safety and Efficacy of Transaortic Transcatheter Aortic Valve Implantation Via Right Mini-Thoracotomy
Source: Interdiscip Cardiovasc Thorac Surg. 2026 Aug 14;41(9):ivag226. doi: 10.1093/icvts/ivag226 (PMC13541777; doi:10.1093/icvts/ivag226)
Supplement: Supplementary file 1 [file ivag226_supplementary_data.docx]

Supplemental Table 1. Case of death during follow-up period

|  | Total cohort | | Matched cohort | |
| --- | --- | --- | --- | --- |
|  | TF | Rt-TAo | TF | Rt-TAo |
|  | n=694 | n=73 | n=63 | n=63 |
| Malignant tumor | 16 (2.3%) | 3 (4.1%) | 0 | 2 (3.2%) |
| Pneumonia | 12 (1.7%) | 6 (8.2%) | 1 (1.6%) | 6 (9.5%) |
| Cerebrobascular death | 6 (0.9%) | 2 (2.7%) | 1 (1.6%) | 2 (3.2%) |
| Heart failure | 18 (2.6%) | 2 (2.7%) | 2 (3.2%) | 2 (3.2%) |
| Aortic aneurysm rupture | 1 (0.1%) | 1 (1.4%) | 0 | 1 (1.6%) |
| Sepsis | 17 (2.4&) | 0 | 1 (1.6%) | 0 |
| Unknown | 12 (1.7%) | 0 | 0 | 0 |
| Liver failure | 1 (0.1%) | 0 | 0 | 0 |
| Aging | 5 (0.7%) | 0 | 0 | 0 |
| Renal failure | 1 (0.1%) | 0 | 0 | 0 |
